# *In Silico* Analysis of the Genes Encoding Proteins that Are Involved in the Biosynthesis of the RMS/MAX/D Pathway Revealed New Roles of Strigolactones in Plants

**DOI:** 10.3390/ijms16046757

**Published:** 2015-03-25

**Authors:** Marek Marzec, Aleksandra Muszynska

**Affiliations:** 1 Department of Genetics, Faculty of Biology and Environmental Protection, University of Silesia, Katowice 40-032, Poland; 2 Department of Physiology and Cell Biology, Leibniz Institute of Plant Genetics and Crop Plant Research (IPK), Gatersleben 06466, Germany; E-Mail: muszynska@ipk-gatersleben.de

**Keywords:** abiotic and biotic stresses, biosynthesis, expression, hormone crosstalk, *in silico* analysis, promoter, strigolactones

## Abstract

Strigolactones were described as a new group of phytohormones in 2008 and since then notable large number of their functions has been uncovered, including the regulation of plant growth and development, interactions with other organisms and a plant’s response to different abiotic stresses. In the last year, investigations of the strigolactone biosynthesis pathway in two model species, *Arabidopsis thaliana* and *Oryza sativa*, resulted in great progress in understanding the functions of four enzymes that are involved in this process*.* We performed *in silico* analyses, including the identification of the *cis*-regulatory elements in the promoters of genes encoding proteins of the strigolactone biosynthesis pathway and the identification of the miRNAs that are able to regulate their posttranscriptional level. We also searched the databases that contain the microarray data for the genes that were analyzed from both species in order to check their expression level under different growth conditions. The results that were obtained indicate that there are universal regulations of expression of all of the genes that are involved in the strigolactone biosynthesis in *Arabidopsis* and rice, but on the other hand each stage of strigolactone production may be additionally regulated independently. This work indicates the presence of crosstalk between strigolactones and almost all of the other phytohormones and suggests the role of strigolactones in the response to abiotic stresses, such as wounding, cold or flooding, as well as in the response to biotic stresses.

## 1. Introduction

Strigolactones are a hormone group that has been intensively studied during the last few years. Although they were originally described as an inductor for the germination of the seeds of parasites [1], they were gradually determined to play a role in the signaling between plants and other organisms, such as fungi [2] or bacteria [3], as well as in plant growth and development. The first reports that indicated that strigolactones were negative regulators of the branching of the aboveground part of plants were published in 2008 [4,5]. Subsequent analyses revealed additional functions of strigolactones in plant growth, which included the regulation of the root system development [6,7], the elongation of the mesocotyl and stem [8,9], secondary growth [10], and shoot gravitropism [11]. Participation in plant growth and development processes appears to be a universal role of strigolactones among different species, including both mono- and dicotyledonous plants (reviewed by [12,13,14]).

Additionally, the role of strigolactones in the adaptation to abiotic stresses, mainly in response to nutrient stress, including phosphorus (P) and nitrogen (N) deficiency, has been proposed [15,16]. It was shown that plants increased the production and the exudation of strigolactones in conditions of P and N starvation [17,18,19,20], which allowed the symbiosis with *arbuscular mycorrhizal* fungi or N-fixing rhizobial bacteria to be enhanced and the development program to be adapted in order to promote the most efficient use of the available resources (reviewed by [21]). The importance of strigolactones in conditions of dehydration stress, including salt and drought stress, has also been proposed. Plants under salt stress increased exudation of strigolactones and additionally strigolactone-deficient or strigolactone-response mutants were hypersensitive to both drought and salt stress [22,23,24].

**Figure 1:**
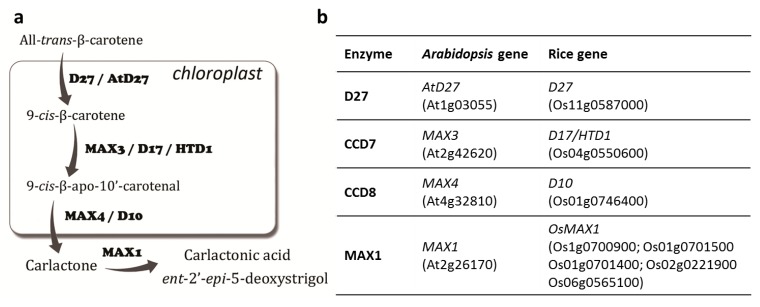
Strigolactone biosynthesis. (**a**) Scheme of the biosynthesis pathway divided into four steps and (**b**) genes encoding proteins that are involved in each stage of this process in *Arabidopsis* and rice.

Significant progress has been made in uncovering the mechanism of strigolactone biosynthesis in the last few years and it now appears that the main players that are involved in this process are known. Strigolactones are carotenoid derivatives that are synthesized in roots that belong to lactones and are composed of four rings (A–D)—a tricyclic lactone core (the ABC part) and a butenolide moiety (the D ring) [25,26]. The C–D part is conserved among the strigolactones that have already been described, while the A–B rings are subjected to modifications, including the substitution of the methyl, hydroxyl and acetyloxyl groups [27,28]. Strigolactone biosynthesis is localized in the chloroplasts, in which three of four enzymes that are already known to be involved in this process are active (Figure 1a). The first one is the carotenoid isomerase D27 (DWARF27), which is an iron-containing protein that is able to convert all-*trans*-β-carotene into 9-*cis*-β-carotene [29]. This carotenoid isomerase is encoded by the *D27* (Os11g0587000) and *AtD27* (At1g03055) genes that were identified in *Oryza sativa* (rice) [30] and *Arabidopsis thaliana* [31], respectively. The product of the activity of the D27 isomerase is immobile and it is a substrate for the next stages of strigolactone production, which are conducted by the carotenoid cleavage dioxygenases (CCDs). The first one, CCD7, is a stereo-specific dioxygenase that cleaves to only 9-*cis*-β-carotene to produce 9-*cis*-β-apo-10'-carotenal, which is subsequently cleaved by CCD8 to produce carlactone (Figure 1a). Genes that encode both CCDs have been identified in many different plant species, including primitive moss [32,33]. Analyses of *Arabidopsis* and rice plants that have mutations in the genes that encode CCD7 (*MAX3*—At2g42620; *D17*/*HTD1*—Os04g0550600) and CCD8 (*MAX4*—At4g32810—*D10*, Os01g0746400) revealed a highly-branched phenotype, which is characteristic for strigolactone mutants that was reversible after treatment with a strigolactone analogue [5,34,35,36,37] (Figure 1b).

The structure of carlactone is similar to the one that has been described for strigolactones, because it consists of a C_19_-skeleton and a C_14_-moiety, which corresponds to the D-ring of strigolactones [29]. Moreover, carlactone exhibits biological activity that is similar to that of strigolactones, including promoting the germination of the seeds of parasites or regulating shoot branching [29,38]. The presence of carlactone in plants as well as its role as a putative intermediate in strigolactone biosynthesis was confirmed for the first time in 2014. Using ^13^C-labeled carlactone, the authors proved the conversion of this compound into (–)-[^13^C]-2'-*epi*-5-deoxystrigol and ^13^C-orobanchol, which are the two main precursors of other strigolactones [39]. This conversion, which includes the oxidation of two carlactone positions followed by dehydrogenation [29], is mediated by the last enzyme from the strigolactone biosynthesis pathway, monooxygenase MAX1, which belongs to the cytochrome P450 family. *MAX1* was originally characterized in *Arabidopsis* (At2g26170) and for a long time its function remained unknown, but grafting experiments have suggested that MAX1 acts downstream of both CCDs [40]. Further analysis revealed five *MAX1* homologues in rice (Figure 1b), but two of them (Os01g0700900, Os01g0701400) were only present in highly tillered rice varieties that were low strigolactone producers (e.g., *cv. Azucena*) and that the deletion of these two *MAX1* homologues on chromosome 1 was associated with the natural variation of strigolactone biosynthesis in rice [41]. In the next published report, the biochemical function of two rice *MAX1* homologues was characterized—Os01g0700900 catalyzes the oxidation of carlactone to produce the first strigolactone *ent-*2'-*epi*-5-deoxystrigol, whereas Os01g0701400 is involved in the production of orobanchol via the hydroxylation of the *ent-*2'-*epi*-5-deoxystrigol, and thus plays a role in the structural diversification of strigolactones [42]. As was mentioned for *Arabidopsis*, only one gene encoding *MAX1* is present, and recently it was proven experimentally that this monooxydase converts carlactone into carlactonoic acid (9-desmethyl-9-carboxy-carlacton) [43]. Additionally, using the strigolactone pathway in *Arabidopsis* that was reconstructed in *Nicotiana benthamiana*, it was shown that MAX1 is not able to produce orobanchol, but only small amount of *epi*-5-deoxystrigol and 5-deoxystrigol [42]. There are no close homologues of *MAX1* in the *Arabidopsis* genome and questions about other enzymes that may be involved in the production of different strigolactones from 5-deoxystrigol in this species remains unanswered.

The new reports on the multiple functions of strigolactones in plant growth and development, as well as in stress responses or crosstalk with other hormones, have indicated that there might still be some uncovered roles of strigolactones in plants. Because the genetic background of the strigolactone biosynthesis pathway seems to be well studied and increased production of these hormones during responses to different stress stimuli has been described, *in silico* analyses of genes encoding proteins that are involved in strigolactone biosynthesis may shed a new light on the roles that strigolactones play in plants. In the presented work, we analyzed the sequences of genes that are involved in strigolactone production in two model species *Arabidopsis* and rice in an attempt to determine new potential functions of strigolactones. 

## 2. Results

### 2.1. Analysis of the Promoter Sequences of the Arabidopsis Genes that Are Involved in Strigolactone Biosynthesis

**Figure 2:**
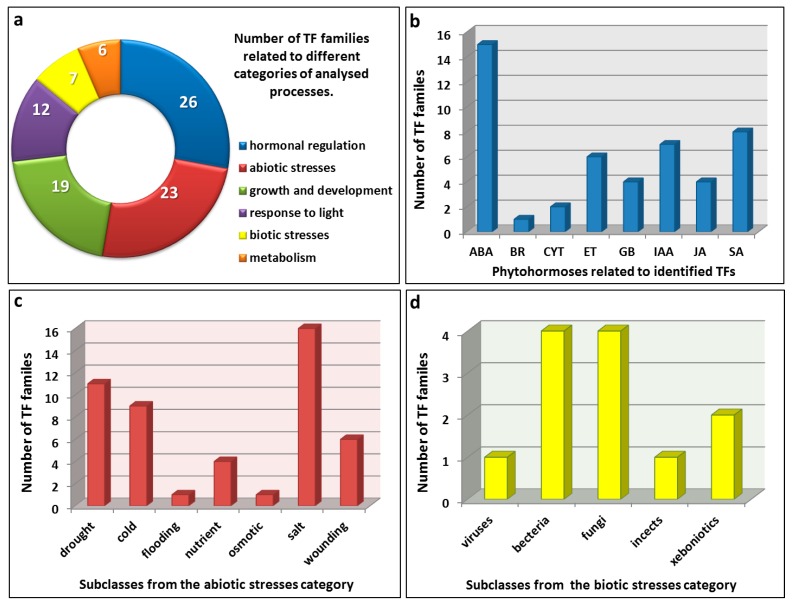
Categories of the processes that are regulated by the TFs that have binding sites in the promoter sequences of genes encoding proteins that are involved in strigolactone biosynthesis. (**a**) The six main categories of the processes that are regulated by the TFs that were identified; (**b**) detailed information about the TFs that are involved in hormonal regulation; (**c**) the number of TF families that are related to different abiotic; and (**d**) biotic stresses.

Analysis of the promoter sequences of four genes (*AtD27*, *MAX1*, *3* and *4*), which encode the proteins that are involved in strigolactone biosynthesis in *Arabidopsis* revealed 55 different motifs for the binding of transcription factors (TFs) (Table 1). The highest number of different TF motifs and their repeats was observed for the promoter of *MAX3* (46 and 143, respectively). A similar number of binding sites for TFs was found in the promoters of *MAX1* and *MAX4* (30 and 34, respectively), but the greatest number of individual binding sites was observed in the *MAX4* promoter in comparison to *MAX1* (131 *vs.* 96). The lowest number of TF motifs was found in the promoter of *AtD27* (26 TF families and 94 motif repeats). Among the TF families that were identified, the greatest number of them were related to hormonal regulation (26), abiotic stresses (23) and plant growth and development (19), whereas the TFs that are involved in the response to light (12), biotic stresses (7) and metabolism (6) were low (Figure 2a). The largest class of *cis*-regulatory elements that were identified, which were related to hormonal regulation, can be divided into specific phytohormone subgroups, such as abscisic acid (15), auxin (7), brassinosteroid (1), cytokinin (2), ethylene (6), gibberellin (4) jasmonic acid (4) and salicylic acid (8) (Figure 2b). Among the TFs that are involved in the responses to abiotic stresses, those related to salt (16), drought (11), low temperatures (9), wounding (6), nutrients (4), flooding (1) and osmotic stress (1) can be distinguished (Figure 2c), while the biotic stresses category can be divided according to pathogens, such as viruses (1), bacteria (4), fungi (4), insects (1) or xenobiotics (2).

**Table 1:** Motifs for TFs and their number, which were found in the promoter sequences of genes encoding proteins in *Arabidopsis* that are involved in strigolactone biosynthesis.

TFs Motifs		*AtD27*		*MAX3*	*MAX4*	*MAX1*	Gene Ontology (GO) Process
AGL3		1		4	4	4	growth and development
AG		3		4	6	4	growth and development
ATHB-1		9		11	14	6	abiotic stresses (salt, nutrients); growth and development; response to light
ATHB-5		9		10	8	8	hormonal regulation (ABA)
ATHB-9		5		7	9	7	growth and development
RAV1-A/RAV1AAT		5		6	1	3	growth and development; hormonal regulation (BR); metabolism
ACGTATERD1		4		10	6	2	abiotic stresses (drought, salt); response to light
ANAERO1-3CONSENSUS		2		1	3	1	abiotic stresses (flooding)
ARR10		5		3	5	2	growth and development; hormonal regulation (CYT)
ARR1AT		7		17	10	6	growth and development; hormonal regulation (CYT); metabolism
ASF1MOTIFCAMV		1		2	2	1	biotic stresses ( bacteria, xenobiotics); hormonal regulation (IAA, SA); response to light
Bellringer		1		1	2	3	growth and development
GATABOX		10		10	18	5	abiotic stresses (nutrients); response to light
GT1CONSENSUS		9		5	8	9	response to light
MYB1AT		3		4	1	3	abiotic stresses (drought, salt)
MYCCONSENSUSAT		6		8	6	9	abiotic stresses (drought, salt); hormonal regulation (ABA)
MYB4		2		2	1	5	abiotic stresses (wounding); hormonal regulation (JA, SA); metabolism
SURECOREATSULTR11		2		1	1	1	abiotic stress (nutrients)
WBOXATNPR1		3		3	3	1	biotic stresses (bacteria, fungi, viruses); hormonal regulation (SA)
CDC5		1		0	1	0	biotic stresses (bacteria, fungi); growth and development
PIF3		0		2	1	0	response to light; hormonal regulation (GB)
ABRE-like		0		2	0	0	abiotic stresses (cold, drought, salt)
ABREATCONSENSUS		0		1	0	0	abiotic stresses (cold, drought, salt); hormonal regulation (ABA)
ABRELATERD1		0		1	1	0	abiotic stresses (cold, drought, salt); response to light
ABRERATCAL		0		0	1	0	abiotic and biotic stresses (induced by Ca^2+^); hormonal regulation (ABA)
ABRE		0		1	0	0	hormonal regulation (ABA)
ACGTABREMOTIFA2OSEM	0		1		0	0	hormonal regulation (ABA)	
AP1	0		1		2	1	growth and development	
ARFAT/ARF	1		1		0	0	growth and development; hormonal regulation (IAA)	
Agamous	0		1		1	3	growth and development	
AtMYB2	0		1		0	0	abiotic stresses (cold, nutrients, salt, wounding); hormonal regulation (ABA, ET, IAA, JA, SA)	
AtMYC2	0		1		1	0	abiotic stresses (wounding); biotic stresses ( fungi, insects); hormonal regulation (ABA, JA, SA); metabolism	
C8GCARGAT	0		4		4	0	growth and development; hormonal regulation (IAA); metabolism	
CGCGBOXAT	0		0		4	0	abiotic stresses (low temperatures, salt, wounding); hormonal regulation (ET, IAA)	
CCA1ATLHCB1/CCA1	1		0		0	1	abiotic stresses (low temperatures, salt); hormonal regulation (ABA, ET, GB, IAA, SA)	
DPBFCOREDCDC3	0		1		0	0	abiotic stresses (drought, low temperatures, salt); biotic stresses (fungi); hormonal regulation (ABA, GB)	
GAREAT	0		1		1	3	hormonal regulation (GB)	
GBF5	0		2		0	0	biotic stresses (xenobiotics); growth and development	
LEAFYATAG	0		0		0	1	growth and development	
LTREATLTI78	0		1		0	0	abiotic stresses (low temperatures)	
LTRECOREATCOR15	0		1		0	0	abiotic stresses (low temperature); response to light	
MYB1LEPR	0		1		0	1	biotic stresses ( bacteria); hormonal regulation (ET)	
MYB2CONSENSUSAT	0		1		0	1	abiotic stresses (drought, salt); hormonal regulation (ABA)	
MYBATRD22	0		1		0	0	abiotic stresses (salt, wounding); hormonal regulation (ABA, ET, IAA, SA)	
MYBCORE	0		1		1	1	abiotic stresses (drought, salt, wounding); hormonal regulation (ABA, ET, IAA, JA, SA)	
MYBPLANT	1		0		1	2	abiotic stresses (drought, salt); hormonal regulation (ABA); metabolism	
MYCATERD1/MYCATRD22	0		1		1	0	abiotic stresses (drought, salt); hormonal regulation (ABA)	
PREATPRODH	0		2		0	0	abiotic stresses (osmotic)	
SITEIIATCYTC	0		0		0	1	growth and development; metabolism	
SORLIP5AT	1		1		0	0	response to light	
SREATMSD	0		1		0	0	growth and development	
SV40COREENHAN	1		1		0	1	response to light	
TBOXATGAPB	1		0		2	0	response to light	
ZDNAFORMINGATCAB1	0		1		0	0	growth and development; response to light	
XYLAT	0		0		1	0	growth and development	

One TF can be involved in different processes and therefore it has been assigned to different categories. The binding sites that are underlined were identified in the promoter sequences of all of the *Arabidopsis* genes that were analyzed; ABA—abscisic acid; BR—brassinosteroid; CYT—cytokinin; ET—ethylene; GB—gibberellin; IAA—auxin; JA—jasmonic acid; SA—salicylic acid.

Special attention should be paid to those of the 19 TF families (34.5% of the total number that were identified) that were found in the promoter sequences of all of the *Arabidopsis* genes that were analyzed (Table 1). Among them, representatives of each of the described categories of biological processes were observed: abiotic and biotic stresses, growth and development, hormonal regulation, metabolism, as well as response to light. The highest number of repeated motifs were identified for GATABOX (43 in total), ATHB-1 (40 in total), ARR1AT (40 in total), ATHB-5 (37 in total) and GT1CONSENSUS (31 in total). The presence of those *cis*-regulatory elements in the promoter sequences of all known genes encoding proteins that are known to be responsible for strigolactone biosynthesis indicate that strigolactones may be involved in the processes that are regulated by the described TFs. On the other hand, the presence of binding sites of TFs that are specific to only individual genes could mean that the different stages of strigolactone production have their own mechanisms of regulation. Interestingly, specific TF motifs were not observed in only the *AtD27* promoter sequence, whereas for the other genes, *MAX1*, *MAX3* and *MAX4*, a different number of specific *cis*-regulatory elements were identified (1, 4 and 2, respectively).

The highest similarity in the *cis*-regulatory motifs was found between *AtD27* and *MAX1* (64.7%; 22 motifs out of 34), but the highest number of binding sites was shared by two genes encoding carotenoid cleavage dioxygenases—*MAX3* and *MAX4* (28 families out of 52; Table 2). The lowest level of similarity in the TFs that can bind in the promoter region was observed for *AtD27* and *MAX3* (44%, 22 TF families out of the 50 that were identified).

**Table 2:** The similarity of TF binding sites that were found in the promoter sequences of the genes encoding proteins in *Arabidopsis* that are involved in strigolactone biosynthesis.

-	*D27*	*MAX3*	*MAX4*	*MAX1*
***D27***	-	44% (22/50)	55.3% (21/38)	64.7% (22/34)
***MAX3***	44% (22/50)	-	53.8% (28/52)	52% (26/50)
***MAX4***	55.3% (21/38)	53.8% (28/52)	-	58.5% (24/41)
***MAX1***	64.7% (22/34)	52% (26/50)	58.5% (24/41)	-

### 2.2. Analysis of the Promoter Region of the Rice Genes that Are Involved in Strigolactone Biosynthesis

Analysis of the promoter region of four rice genes encoding proteins involved in strigolactone biosynthesis revealed binding sites for 18 TFs families, whereof six were present in each promoter sequence (Table 3). Some of the rice motifs, like AMYBOX1 or CGACGOSAMY3, could not be assigned to the one of previously established groups of biological processes and in this case the descriptive information about their function was included. Among TFs that can interact with all of the genes that were analyzed were representatives of all of the biological processes: abiotic and biotic stresses, growth and development, hormonal regulation, metabolism and response to light were present. However, their number was lower in comparison to those identified for *Arabidopsis* genes. Interestingly, almost all of TF motifs described for *D27* (6 from 7) and for *OsMAX1* (6 from 9) were shared with other genes involved in strigolactone biosynthesis in rice (Table 3). The most frequently occurring *cis*-regulatory elements represented in all genes that were analyzed were: WRKY71OS (30 in total), GT1CONSENSUS (28 in total) and GATABOX (25 in total), which are related to the response to abiotic stresses, hormonal regulation and the response to light.

Some *cis*-regulatory elements were observed in the promoter sequences of all of the *Arabidopsis* and rice genes that were analyzed (GATABOX, GT1CONSENSUS) and additionally those binding sites were repeated several times in each gene of both species.

**Table 3:** TF motifs found in the promoter sequences of rice genes encoding proteins that are involved in strigolactone biosynthesis.

TF Motifs	*D27*	*D17*/*HTD1*	*D10*	*MAX1*	Gene Ontology (GO) Process
BIHD1OS	2	2	1	4	biotic stresses (fungi)
GATABOX	10	7	3	5	abiotic stresses (nutrients); response to light
GT1CONSENSUS	7	12	2	7	hormonal regulation (SA); response to light
PYRIMIDINEBOXOSRAMY1A	1	2	1	2	hormonal regulation (GB); growth and development; sugar repression
SITEIIATCYTC	1	2	2	2	growth and development; metabolism; Relative to cytochrome, oxidative phosphorylation
WRKY71OS	6	6	6	12	biotic stresses (pathogens); hormonal regulation (GB)
ABREOSRAB21	0	1	1	0	hormonal regulation (ABA); abiotic stresses (osmotic)
ACGTABOX	0	2	6	0	growth and development; sugar repression
ANAERO1-3CONSENSUS	0	1	0	1	abiotic stresses (flooding)
ARFAT	0	1	0	0	growth and development; hormonal regulation (IAA)
AMYBOX1	0	0	1	0	Conserved sequence found in 5'-upstream region of alpha-amylase gene
E2FCONSENSUS	0	1	0	0	growth and development
CAREOSREP1	0	0	1	0	hormonal regulation (GB)
CGACGOSAMY3	0	0	4	0	Conserved sequence found in 5'-upstream region of alpha-amylase gene
GARE1OSREP1	0	0	2	0	hormonal regulation (GB)
HEXMOTIFTAH3H4	0	1	0	2	hormonal regulation (IAA, SA); metabolism
TATABOXOSPAL	0	1	0	1	abiotic stresses (salt); hormonal regulation (ET, GB, IAA, JA, SA)
TATCCAOSAMY	2	0	0	0	abiotic stresses ( nutrients); hormonal regulation (GB); found in alpha-amylase promoters of rice

One TF can be involved in different processes and therefore it has been assigned to different categories. The binding places for the TFs that are underlined were observed in the promoter sequences of all of the rice genes that were analyzed; ET—ethylene; GB—gibberellin; IAA—auxin; JA—jasmonic acid; SA—salicylic acid.

### 2.3. Identification of miRNA Target Sites in the mRNAs of Genes from the Strigolactone Biosynthesis Pathway

At least one target site for eight different miRNAs were identified in *MAX1*, *MAX3* and *MAX4*. In *MAX3* and *MAX4* one target site, which was complementary to nine different miRNAs, was identified, whereas for *MAX1*, two target sequences that were recognized by 20 miRNAs were identified. The target sequences for seven different miRNAs (ath-miR165b; ath-miR166a-g) were found in the mRNA of all three *MAX* genes. Additionally, the mRNA of *MAX1* and *MAX3* contains sequences that were recognized by the miRNAs that were specific only to them (Table 4).

A large number (69) of target sequences for different miRNAs were identified in the mRNA of four rice genes encoding proteins of the strigolactone biosynthesis pathway. Among them, only three were present in all of the genes: osa-miR444, osa-miR528 and osa-miR531. However, osa-miR531 was also annotated as repetitive sequences [44] and this was the reason why it was excluded from a detailed analysis (Table 4). Additionally, mRNA of *D17*/*HTD1*, *D10 and OsMAX1* were recognized by the miRNAs that are specific only for them (17, 18 and 4 miRNAs, respectively).

**Table 4:** Target sites for the miRNA that was found in the mRNA of *Arabidopsis* and rice genes encoding proteins that are involved in the strigolactone biosynthesis.

miRNA	Position of Target Sites			
	*Arabidopsis* Genes			
	*AtD27*	*MAX3*	*MAX4*	*MAX1*
**ath-miR156g**	-	-	-	302–323
**ath-miR165a**	-	-	950–971	842–861
**ath-miR165b**	-	1469–1493	950–971	842–861
**ath-miR166a-g**	-	1469–1493	951–971	842–861
				1280–1301
**ath-miR395b,c,f**	-	-	-	336–357
**ath-miR401**	-	1701–1725	-	-
**-**	**Rice Genes**			
	***D27***	***D17*/*HTD1***	***D10***	***OsMAX1***
**osa-miR444**	47–70	557–581	172–154	458–478
			1055–1076	
		363–380	221–239	
			1292–1318	
**osa-miR528**	14–33	363–380	1192–1214	331–351
		864–885		1113–1138

### 2.4. Gene Expression after Hormone Treatment and during Responses to Abiotic Stresses

The effect of different hormone treatments on gene expression was checked for all of the *Arabidopsis* genes that are responsible for the strigolactone biosynthesis pathway. There is no universal response in the expression of all of the genes that is induced by abscisic acid, auxin, brassinolide, cytokinin, ethylene, gibberellin acid or methyl jasmonate. Moreover, for some of the genes that were analyzed, the opposite effect (induction or repression) was observed at different time points during treatment with the same hormone (Table 5). However, in some cases the same effect was caused by a hormone at all of the time points, *i.e.*, the induction of *MAX3* was observed during ethylene treatment; abscisic acid, auxin and brassinolide increased the expression of *MAX4*; the transcription of *MAX1* was enhanced by brassinolide and was inhibited by auxin, cytokinin, gibberellin acid and methyl jasmonate treatment (Table 5). 

**Table 5:** Relative level of *Arabidopsis* genes expression in roots after treatment with different hormones.

Treatment	Time Point	log2 Ratio (Sample Signal/Control Signal)			
		*AtD27*	*MAX3*	*MAX4*	*MAX1*
Abscisic acid (10 µM)	0.5 h	0.01	−0.27	0.23	−0.15
	1 h	0.44	0.04	0.23	−0.39
	3 h	−0.56	0.83	0.23	0.05
Auxin (IAA 1 µM)	0.5 h	0.15	−0.19	0.3	−0.04
	1 h	−0.22	−0.54	1.34	−0.38
	3 h	−0.24	0.24	0.58	−0.52
Brassinolide (10 nM)	0.5 h	0.56	−0.35	1.05	0.07
	1 h	−0.05	−0.7	1.07	0.04
	3 h	−0.3	0.1	0.12	0.15
Cytokinin (zeatin 1 µM)	0.5 h	−0.1	−0.05	−0.04	−0.43
	1 h	0	−0.03	0.72	−0.76
	3 h	−0.33	0.37	−0.39	−0.45
Ethylene (ACC 10 µM)	0.5 h	0.45	0.06	0.8	−0.04
	1 h	0.34	0.04	0.67	−0.07
	3 h	−0.63	0.55	−0.26	0.09
Gibberellin acid (1 µM)	0.5 h	0.13	−0.29	−0.08	−0.11
	1 h	0.28	−0.15	0.01	−0.25
	3 h	−0.17	0.44	−0.34	−0.19
Methyl jasmonate (10 µM)	0.5 h	0.53	0.1	0.48	−0.13
	1 h	−0.37	−0.2	0.37	−0.21
	3 h	−1.2	0.22	−0.2	−0.05

Data for seven-day-old seedlings of *Arabidopsis*, Columbia-0 ecotype, which were obtained from the eFP Browser [45,46] and PathoPlant database [47,48]. The mean values of the log2 ratio for two biological replicates were present; ACC (1-aminocyclopropane-1-carboxylic acid)—an ethylene precursor; IAA (indole-3-acetic acid)—an active form of auxin.

The expression of rice genes was investigated in the case of treatment with abscisic acid, auxin, brassinolide, cytokinin, gibberellin acid or jasmonic acid. The relative levels of *D17*/*HTD1*, *D10* and *OsMAX1* expression changed under treatment with the same hormones; and moreover, some of the hormones diversely regulated the expression of individual genes, depending on the time. An equivalent treatment effect was observed for *MAX1* whose expression was induced by abscisic acid, brassinolide or cytokinin and repressed by jasmonic acid; the expression of *D10* after treatment with cytokinin and jasmonic acid decreased, while a lower expression level of *D17*/*HTD1* in comparison to control conditions was observed after treatment with brassinolide (Table 6).

**Table 6:** Relative level of rice genes expression in roots after treatment with different hormones.

Treatment	Time Point	log2 Ratio			Treatment	Time Point	log2 Ratio		
		*D17/HTD1*	*D10*	*MAX1*			*D17/HTD1*	*D10*	*MAX1*
**Abscisic acid (50 µM)**	0.25 h	0.10	−0.07	0.07	**Cytokinin (zeatin 1 µM)**	0.25 h	−0.31	−0.37	0.03
	0.5 h	−0.06	−0.22	0.30		0.5 h	0.26	−0.39	0.71
	1 h	−0.31	−0.15	0.89		1 h	−0.12	−0.28	0.92
	3 h	0.26	0.06	1.43		3 h	−0.20	−0.24	1.06
	6 h	0.57	0.24	2.32		6 h	−0.17	−0.11	0.79
**Auxin (IAA 10 µM)**	0.25 h	0.21	0.05	0.21	**Gibberellin acid (10 µM)**	0.25 h	−0.02	−0.44	−0.34
	0.5 h	−0.35	−0.17	0.09		0.5 h	−0.27	−0.06	−0.66
	1 h	−0.26	−0.38	0.33		1 h	−0.01	−0.12	−0.41
	3 h	1.11	−0.06	0.66		3 h	0.23	−0.13	−0.63
	6 h	0.29	−0.09	−0.02		6 h	−0.12	0.38	0.07
**Brassinolide (1 µM)**	0.25 h	−0.43	0.02	0.15	**Jasmonic acid (100 µM)**	0.25 h	−0.26	−1.65	−0.17
	0.5 h	−0.13	−0.13	0.22		0.5 h	0.90	−1.01	−0.30
	1 h	−0.44	−0.25	0.13		1 h	0.79	−0.69	−0.59
	3 h	−0.19	−0.02	0.25		3 h	2.46	−0.82	−0.70
	6 h	−0.15	0.02	0.35		6 h	2.77	−0.85	−0.87

Data for seven-day-old seedlings of rice, cv. Nipponbare, according to the RiceXPro database [49,50]. Mean values of log2 ratio for three biological replicates were present; IAA (indole-3-acetic acid)—an active form of auxin.

The relative expression levels of the gene-encoding proteins that are involved in strigolactone biosynthesis in *Arabidopsis* were also analyzed under different abiotic stresses, such as drought, cold, osmotic stress, wounding, salt, heat, UV-B, genotoxic or oxidative stress. None of stress factors that were tested had the same influence in regards to increasing or decreasing the expression of all of the genes from the strigolactone biosynthesis pathway. Moreover, at different time points under the same stress the expression of the genes that were analyzed was modulated in two different ways, which was similar to that observed for the hormone treatment. However, the response of individual genes to some of stress factors was the same: the expression of *AtD27* decreased under salt, genotoxic and UV-B stress, while the expression of *MAX3* was induced under wounding, heat, UV-B and genotoxic stress conditions (Table 7).

**Table 7:** Relative level of *Arabidopsis* gene expression under the response to the different stresses.

Stress	Time	*AtD27*	*MAX3*	*MAX4*	*MAX1*	Stress	Time	*AtD27*	*MAX3*	*MAX4*	*MAX1*
**Cold**	0.5 h	0.25	−0.3	−0.47	0.08	**Drought**	0.25 h	−1.01	0.33	−0.61	0.26
	1 h	−0.08	0.29	−0.1	0.37		0.5 h	−0.15	−0.44	−0.28	−0.25
	3 h	0.24	0.14	0.05	0.04		1 h	−0.7	0.76	−0.08	0.62
	6 h	0.15	−0.93	0.33	0.35		3 h	0.03	0.9	0.08	0.01
	12 h	−0.22	−0.82	0.31	−0.87		6 h	0.21	0.57	−0.09	0.49
	24 h	−0.04	−0.13	−0.28	−2.27		12 h	0	0.36	0.14	−0.03
**Osmotic**	0.5 h	0.23	−0.24	−0.03	−0.28		24 h	−0.55	0.19	0.16	−0.09
	1 h	−0.05	0.62	−0.13	−0.04	**Wounding**	0.25 h	−1.36	0.8	−0.09	−0.06
	3 h	−0.11	1.06	−0.01	−0.15		0.5 h	0.13	0.19	−0.14	−0.02
	6 h	0.33	0.72	0.73	0.53		1 h	−0.31	0.52	−0.33	0.39
	12 h	−0.82	1.35	1.6	0.39		3 h	0.38	0.42	−0.24	0.08
	24 h	−0.67	0.97	1.4	0.71		6 h	−0.49	0.46	−0.11	0.51
**Salt**	0.5 h	−0.02	0.24	−0.14	0.21		12 h	−1.44	0.75	−0.34	−0.14
	1 h	−0.54	0.66	−0.34	0.13		24 h	−0.51	0.38	0.24	−0.05
	3 h	−0.35	0.43	−0.03	−0.46	**Heat**	0.25 h	−0.09	0.45	0.23	0.08
	6 h	−0.36	−0.6	0.38	−0.61		0.5 h	0.23	0.21	0.03	0.49
	12 h	−0.94	0.18	1.24	0.27		1 h	−0.2	1.17	−0.86	0.09
	24 h	−0.61	0.13	0.42	−0.15		3 h	−0.02	1.65	−0.49	−0.55
**Genotoxic**	0.5 h	−0.23	0.15	−0.15	0.01		4 h	0.05	0.2	0.53	0.46
	1 h	−1.16	0.64	−0.19	0.14		6 h	−0.55	0.47	−0.18	−0.15
	3 h	−0.59	0.18	−0.45	−0.1		12 h	−0.96	0.95	0.61	0.75
	6 h	−0.51	0.01	0.18	0.12		24 h	0.2	0.18	0.12	0.19
	12 h	−0.95	0.16	0.46	0.22	**UV-B**	0.25 h	−0.34	0.24	−0.25	−0.22
	24 h	−0.11	0.1	0.44	0.09		0.5 h	−0.11	0.64	−0.11	0.34
**Oxidative**	0.5 h	−0.19	0.18	−0.25	0.01		1 h	−0.68	1.15	−0.96	0.49
	1 h	−0.47	0.42	−0.23	0.22		3 h	−0.26	0.56	−1.01	−0.32
	3 h	0.5	0.68	−0.03	−0.24		6 h	−0.36	0.16	−0.55	0.13
	6 h	−0.52	0.41	−0.34	0.04		12 h	−0.57	0.29	−0.22	0.22
	12 h	−0.4	−0.1	0.48	0.06		24 h	−0.53	0.2	0.07	−0.12
	24 h	−0.27	0.42	0.55	0.51		-	-	-	-	-

Data for seven-day-old seedlings of *Arabidopsis*, Columbia-0 ecotype, which were obtained by Kilian and co-workers [51]. Mean values of log2 ratio for two biological replicates were present.

## 3. Discussion

### 3.1. Regulation of the Expression of Genes that Are Responsible for Strigolactone Biosynthesis via TFs and miRNAs

To the best of our knowledge, this is the first report describing which TFs may regulate the strigolactone biosynthesis; however, the role of some TFs in the regulation of individual genes related to strigolactone biosynthesis has been postulated previously [52]. TFs play a crucial role in the coordination of plant growth and development, as well in the response to different stresses because they are able to regulate the spatial expression of the many different genes that are contained in the promoter region motifs that are recognized by TFs [53]. Based on the experimental data that describes the role the regulation of gene expression in model species [54,55], it is possible to screen the promoter region of any gene to identify which TFs are able to influence its expression [56,57,58]. Knowledge about which TFs can activate the gene expression allows the function of the gene that was analyzed in plant development program or response to external factors to be predicted. 

Plant hormones are involved in many processes during a plant’s life and the genes encoding proteins that are responsible for the biosynthesis or signaling pathway are under the control of different TFs [59,60]. Based on the fact that the binding site for a specific TF is present in the promoter region of all of the genes that are responsible for the biosynthesis pathway of strigolactones, we assumed that strigolactones may be involved in the processes that are regulated by this TF. In the case of *Arabidopsis* genes, the 19 motifs of *cis*-regulatory elements were present in the promoter region of all four of the genes that were analyzed and most of them were identified in replicates (Table 1). Among them, the majority is related to processes that were already described as being regulated by strigolactones, such as: plant growth and development (AGL3, AG, ATHB-9, Bellringer) [14]; response to nutrient stress (ATHB-1, GATABOX, SURECOREATSULTR11) [21,61]; response to light (GT1CONSENSUS) [62,63]; or very recently some have been postulated to be involved in the response to drought (like ACGTATERD1, MYB1AT) [23,24]. However, some of the TFs that were identified in the promoter region of all of the *Arabidopsis* genes that were analyzed are involved in processes that have not yet been linked to strigolactones. The presence of the WBOXATNPR1 and ASF1MOTIFCAMV motifs indicates that the strigolactone biosynthesis pathway may be induced during a response to biotic stresses. Both *cis*-regulatory elements are bound by the TFs that are induced by salicylic acid [64] and it was confirmed that both TFs play a role in a plant’s defense reaction against viruses, bacteria or fungi [65,66]. Moreover, an analysis of the genes encoding strigolactone biosynthesis pathway in rice revealed the presence of BIHD1OS and WRKY71OS motifs, which are related to defense mechanisms against fungi and other pathogens, in each promoter region [67,68]. This possible new role of strigolactones might be explained by the known connection between *arbuscular mycorrhizal* symbiosis that is regulated via strigolactones and plant responses to biotic stresses (reviewed by [69]).

**Figure 3:**
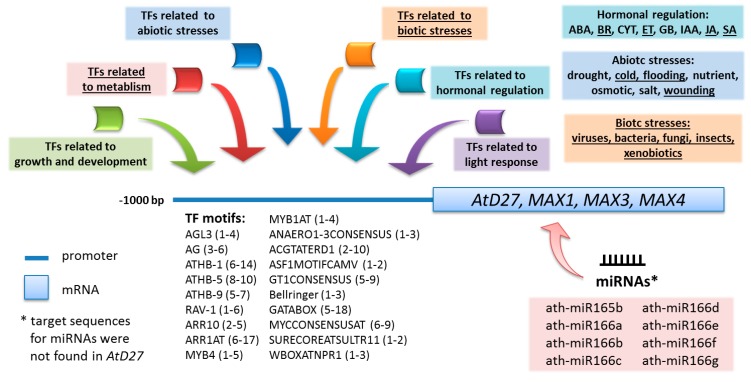
Regulation of the expression and post-transcriptional activity of *Arabidopsis* genes that encode the different stages of strigolactone production; common mechanisms were present for all of the genes that were analyzed and the new predicted functions of this phytohormone class were underlined.

The second unexpected process that might be linked to strigolactones that is based on an *in silico* analysis is their participation in the response to flooding (because of the presence of the ANAERO1-3CONSENSUS motifs). These motifs were found in the promoter region of anaerobically induced genes that are involved in the fermentative pathway [70] and were present in all of the *Arabidopsis* genes that are known to be responsible for strigolactone biosynthesis as well as in individual rice genes (*D17*/*HTD1* and *MAX1*). The response to flooding mainly covers cell protection against cytosolic acidification and the accumulation of reactive oxygen species and toxins as well as the activation of the enzymes in the fermentation pathway [71,72]. To the best of our knowledge, there is no literature data to date that indicates a link between strigolactones and a resistance to flooding, and therefore we may speculate that the part of the flood-response that covers plant growth may be regulated by strigolactones. However, our analysis also revealed that a few of the TFs that are involved in the metabolic processes have their binding sites in promoter regions of the genes that were analyzed: in rice these were mainly related to alfa-amylases and sugar (*i.e.*, PYRIMIDINEBOXOSRAMY1A, AMYBOX1, CGACGOSAMY3), whereas in *Arabidopsis* they were mainly related with fatty acid beta-oxidation, oxidative phosphorylation or polyamine catabolic process (*i.e.*, ARR1AT, MYB4, SITEIIATCYTC), which may be related to the response to flooding (Figure 3).

The gene activity on the post-transcriptional level can be controlled *i.e.*, by miRNAs. miRNAs are short (21–24 nucleotides) molecules, which based on the sequence complementarity join to mRNA, thus resulting in the degradation or repression of translation (reviewed by [73,74]). The miRNAs that repress TFs have the greatest impact on gene expression because through the inactivation of an individual TF, they influence the expression of all of the genes that are regulated via this TF. However, miRNA can not only inhibit the production of TFs, but also other proteins, *i.e.*, from hormone biosynthesis or signaling pathways, such as the auxin receptor that is encoded by *TIR1*/*AFB2* [75]. Moreover, it was shown that miRNAs play a main role in the response to different external factors, such as drought [76] or wounding [77]. In the mRNAs of the genes that were analyzed, target sequences that were recognized by the same miRNAs were present; in *Arabidopsis* for *MAX1*, *-3* and -*4*, and in rice for all of the genes. This is a clear indication of the presence of at least one universal post-transcriptional mechanism that is able to inhibit the entire pathway of strigolactone biosynthesis. Moreover, some of the miRNAs that were identified regulate the activity of not only the *MAX1*, *-3* and -*4* genes, but also TFs, which induces the expression of some of the genes that were investigated, such as miR165b whose target gene is *ATHB9* [78]. Other miRNAs, such as miR166a-g, which recognized the mRNA of *MAX1*, *-3* and *-4*, have been described as being involved in the regulation of the initiation of the axillary meristem and leaf development, which confirms a role of strigolactones in plant growth that was already known [79]. However, except the miRNAs that repress all of the genes from the strigolactone biosynthesis pathway, some others have only been specific to the particular mRNAs that were analyzed, which indicates that the different stages of strigolactone production have their own mechanisms of post-transcriptional regulation. 

### 3.2. Expression of Strigolactone Biosynthesis Genes after Hormone Treatment and under Stress Conditions

Analysis of the TFs that induced the expression of the strigolactone biosynthesis genes showed a large number of TF families that are related to hormonal regulation. In the case of the *Arabidopsis* genes (*AtD7*, *MAX1*, *MAX3* and *4*), TFs that are associated with almost all of phytohormones were identified, including abscisic acid, auxin, brassinosteroid, cytokinin, ethylene, gibberellin, jasmonic acid or salicylic acid, whereas the analysis of the rice genes encoding proteins that are involved in strigolactone production revealed that their expression can be regulated via the TFs that are related to auxin, ethylene, gibberellin, jasmonic acid or salicylic acid. The question is whether other hormones may really affect strigolactone production.

Within the last year, a great deal of progress has been made in uncovering the relationships between strigolactones and other hormones; however, many interactions remain unexplained (reviewed by [80]). The best known is the crosstalk between strigolactones, auxin and cytokinin during axillary bud outgrowth. The growth of an axillary bud depends on the export of auxin and strigolactones that can inhibit its polar transport via the disorganization of the arrangement of PIN proteins [81,82], which means that strigolactones and auxin act together to arrest growth of an axillary bud, whereas cytokinin works antagonistically to them and promotes the outgrowth of a bud [83]. Several reports have indicated that auxin may induce the expression of *MAX3* and *MAX4* in *Arabidopsis* [84], as well as its homologues in other species, like pea or rice [85,86]. However, an analysis of available microarray data revealed that the auxin treatment of *Arabidopsis* and rice seedlings affects the expression of the different components of strigolactone biosynthesis pathway differently, including *MAX3* and *MAX4*, and additionally that this effect depends on the time of treatment (Table 4 and Table 5). Similar results were obtained for other hormones; none of the hormones that were analyzed universally affected the entire pathway of strigolactone synthesis in either of the species that were investigated; however, each of them somehow changed the relative expression levels of all of the genes in *Arabidopsis* and rice that were analyzed. This is a strong confirmation of the roles of the previously identified TFs that are related to hormone regulation.

One explanation of different effects of hormone treatment on the expression of specific genes from the strigolactone production pathway might be the fact that the different stages of strigolacone production have their own mechanisms of regulation and additionally that strigolactones are tissue-specific [87] or that their functions depend on additional external conditions [13]. The diverse expression of the genes that are involved in strigolatone biosynthesis or the diverse level of the compounds from the strigolactone biosynthesis pathway in a plant’s response to external factor has already been already described. For example, an analysis of two *MAX1* homologues in rice revealed that the expression of *Os01g0700900* is induced under P starvation, whereas *Os01g0701400* expression remained unchanged under the same conditions [41]. This means that of the two enzymes that are involved in the consecutive stages of strigolactone biosynthesis, the production of *ent-*2'-*epi*-5-deoxystrigol and orobanchol, respectively [42], only one plays a role in the response to nutrient stress. Moreover, a measurement of carlactone and 5-deoxystrigol levels in *Arabidopsis* and rice plants that were growing in low P conditions demonstrated that only the level of 5-deoxystrigol was significantly elevated, but that the carlactone level decreased slightly [39,88]. The simplest explanation of experimental and *in silico* results that are obtained for strigolactone biosynthesis genes expression and the level of strigolactone compounds under different stresses or after hormone treatment is assumed to be that some strigolactone metabolites are stored by plants and constitute a reservoir for the rapid synthesis of their derivatives, which is mediated by individual enzymes in response to stress or hormone treatment.

### 3.3. In Silico Analysis for the Prediction of New Roles of Strigolactones in Plants

The results that were obtained during an *in silico* analysis of genes encoding proteins that are involved in strigolactone biosynthesis revealed new, as yet unconfirmed, roles of strigolactones in a plant’s response to biotic and abiotic stresses, as well as new crosstalk between strigolactones and other hormones. Moreover, the studies that are presented confirm the contribution of strigolactones in response to dehydration stresses, which have been described very recently [23,24]. Both reports point out the positive role of strigolactones during drought and salt stresses. Ha and co-workers showed that both strigolactone-deficient (*max3-11*, *max4-7*) and signaling (*max2-3*) mutants were hypersensitive to dehydration and concluded that strigolactones modulate the stress response to drought via abscisic acid-dependent and abscisic acid-independent pathways in *Arabidopsis* [24]. The studies that were carried out by Bu and co-workers indicate that only the strigolactone-response *Arabidopsis* mutants (*max2-1*, *max2-2*) are hypersensitive to drought, but that the strigolactone-deficient ones (*max1*, *max3*, *max4*) do not differ in comparison to the wild-type, which means that strigolactones are not involved in the abscisic acid response pathway [23]. These differences might result from the different methods that are used for the induction of drought stress and need to be clarified through additional investigations; however, they also indicate that the role of strigolactones in the stress response is heterogeneous and may be restricted only to the biosynthesis/signaling part or to the individual stages of one of those processes. Hence, the results that are presented in this paper, which did not show a universal response in the expression levels of all genes from strigolactone biosynthesis under different treatments, remain consistent with the experimentally generated data. Moreover, in our opinion the link between TFs and the fact that they may affect the transcription level of the genes that were analyzed and change their expression under the different conditions that are related to described TFs is a strong indication that strigolactones play many different roles in plants, which have still not been confirmed experimentally (Figure 3).

## 4. Experimental Section

### 4.1. Promoter Analysis

The 1000 bp long promoter sequences of *Arabidopsis* genes (At1g03055; At2g42620; At4g32810 and At2g26170) were obtained using the PlantPAN analytical resource [89,90], whereas the same length of promoter sequences of rice genes (Os11g0587000; Os04g0550600; Os01g0746400 and Os01g0701500) were found in Rice Annotation Project Database [91]. The promoter sequences that were obtained were analyzed using three databases: AGRIS [92,93], JASPAR [94,95] and PLACE [96,97] in order to find the TF motifs. The list of TF families that were obtained for each gene were compared to each other in order to predict any TFs that were common for the *Arabidopsis* and rice genes that are involved in strigolactones biosynthesis.

**Figure 4:**
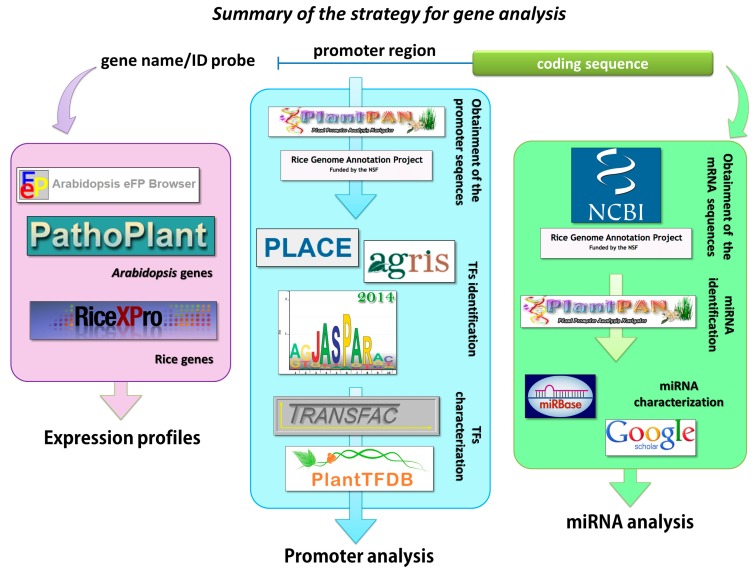
Scheme presented the strategy and tools used during analysis. Detailed description was given in the Experimental Section.

TFs were assigned to the different processes based on the information that are available in the TRANSFAC Database [98,99], the PlantTF Database [100,101] and in the literature. Six main groups of biological processes were created: abiotic stresses, biotic stresses, growth and development, hormonal response, metabolism and response to light (Figure 4).

### 4.2. Identification of the Sequences that Were Recognized by miRNA 

The sequences of mRNA for all of the *Arabidopsis* and rice genes were obtained from the NCBI GeneBank and analyzed using the PlantPAN Database [89,90] in order to identify the target sites for miRNA. The Information about possible role of miRNA in plants was collected via miRBase [102,103] and data from the literature (Figure 4).

### 4.3. Expression Profiles

The expression of *Arabidopsis* genes was analyzed using the eFP Browser tool [45,46] and the PathoPlant database [47,48]. For *AtD27*, *MAX3* and *MAX1*, their TAIR ID was used to explore the databases, whereas for *MAX4* its Affymterix Probe ID was used (253398_at). The data for abiotic stresses was obtained from the results that were published by Kilian and co-workers [51] and included cold, osmotic, salt, genotoxic, oxidative, drought, wounding, heat and UV-B stress that had been tested on 18-day-old seedlings of the wild-type Columbia-0 ecotype. The data for hormonal treatment came from the eFP Browser and PathoPlant resources and they include the response to abscisic acid, auxin, cytokinin, ethylene, methyl jasmonate, gibberellin and brassinolide [45,47]. Those data were collected for the roots of seven-day-old seedlings of *Arabidopsis thaliana*, Columbia-0 ecotype.

Only three rice gene-encoding proteins that are responsible for the strigolactone biosynthesis pathway were assigned to the probes that were analyzed under microarray experiments—*D17*/*HTD1*, *D10* and *OsMAX1* (Os01g0701500). The expression in the roots was investigated under hormonal treatment using abscisic acid, auxin, brassinolide, cytokinin, gibberellin or jasmonic acid and the data was obtained from the RiceXPro Database [49,50]. Analysis was carried out on the roots of seven-day-old seedlings of *Oryza sativa* L. *japonica* *cv.* Nipponbare (Figure 4).

The relative expression level of the genes that were analyzed is presented as the log2 fold change between the control conditions and the different treatment or stress factors. Data for genes of the same species was compared only when it was obtained in the same experiment with at least two biological replicates.

## 5. Conclusions

The results that are presented indicate that strigolactones may be involved in plant responses to many different abiotic stresses (flooding, wounding, cold) and biotic stresses (attack of viruses, bacteria, fungi or insects) that have not been linked to this group of hormones before. Additionally, strigolactone production can be regulated in many different ways since genes that are involved in this process are under the regulation of the TFs that are related to almost all classes of plant hormones. During the analysis of the genes in both species, it was possible to distinguish the group of TFs that is able to regulate the expression of all of the genes from individual species that were analyzed, as well as TFs that bind the promoters of all of the genes that have already been identified for *Arabidopsis* and rice. Additionally, the universal mechanism of the post-transcriptional regulation of gene activity via miRNAs was also present in both species. Taken together, these results indicate that up- or down-regulation of all of the components in the strigolactone biosynthesis pathway are necessary in some aspects of plant growth or adaptation to environmental stimuli. However, an even more important conclusion comes from the observation that each gene also has an individual mechanism for regulation that is related to TFs, as well as miRNAs. These observations are consistent with experimental data that has already been published and that indicate that the role of strigolactones in stress response or plant developmental program may be restricted only to the individual stages of their production.

We believe that data that is presented will facilitate the planning of future experiments that will allow our assumptions about the new roles of strigolactones in plants, especially those that are related to the responses to biotic stresses or metabolism that have not been postulated before, to be confirmed.
